# Calcinosis Universalis of the Elbow: A Rare Case with Classical Presentation

**DOI:** 10.1155/2015/505420

**Published:** 2015-10-22

**Authors:** Sebastian Philipp Boelch, Thomas Barthel, Sascha Goebel, Maximilian Rudert, Piet Plumhoff

**Affiliations:** ^1^Department of Orthopaedic Surgery, The University of Würzburg, König Ludwig Haus, Brettreichstrasse 11, 97074 Würzburg, Germany; ^2^Helios Klinik Volkach, Schaubmühlstrasse 2, 97332 Volkach, Germany

## Abstract

Juvenile Dermatomyositis (JDM) is a rare autoimmune disease in children and adolescents. In these patients calcinosis might be the most characteristic symptom. However there are only few reported cases of intramuscular calcinosis in Dermatomyositis. We report a case of calcinosis universalis (CU) of the elbow in JDM successfully treated with broaching. The patient, a 24-year-old woman, suffered from a long history of JDM. On examination she presented with a fistula lateral to the olecranon and pain of the right elbow joint. Plain X-rays displayed a diffuse pattern of multiple periarticular, subcutaneous, and intramuscular calcifications. The patient underwent surgery for histological and microbiological sampling as well as broaching. Intraoperatively sinus formation and subfascial hard calcium deposition were found. Due to the risk of collateral tissue damage, incomplete broaching was performed. A local infection with *Staphylococcus* was diagnosed and treated with antibiotics. On six-week and 30-month follow-up the patient was free of pain and had very good function. Calcifications on standard radiographs had almost resolved entirely. This case report gives a summary on calcinosis in Dermatomyositis and adds a new case of recalcitrant CU to the literature. Broaching surgery proved to be a reliable treatment option in symptomatic calcinosis.

## 1. Introduction

The incidence of juvenile Dermatomyositis (JDM) varies between two and seven per 1000000 per year [1, 2]. JDM presents with symmetric weakness of proximal muscles and impaired general condition with malaise and fatigue. This condition is typically accompanied by dermal alterations and rash.

The association of subcutaneous and soft tissue calcific deposits with JDM is estimated from 20 to 70% of the cases [2–4]. Little is known about the pathogenesis of calcinosis, but chronic inflammation and repeated trauma releasing calcium and inducing mineralization may play a role. The deposits are characterized by insoluble calcium salts, previously specified as calcium hydroxylapatite [4]. They are found in soft tissues either subcutaneously or in deeper layers. Calcinosis may present in a localized or a disseminated pattern in the patient's body. Typically it occurs in the fingertips or the extensor aspects of the joints. Mostly manifestation can be observed periarticularly or in regions of repeated trauma [5]. Clinical features of calcinosis associated with JDM are muscular atrophy, joint contractions, ulceration, local infection, and pain [5–7]. Although there is no gold standard, diagnosis can be made by patient history, clinical presentation, and X-ray films, which visualise the calcific lesions. Additional imaging, laboratory testing, and histological examination may help to confirm diagnosis and to discriminate against other aetiologies such as Hypervitaminosis D, nephropathy, hyperparathyroidism, or progressive osseous heteroplasia [8]. Calcinosis associated with rheumatic diseases is referred to as dystrophic calcinosis. Dystrophic calcinosis is subgrouped dependent on the pattern of affected tissue into calcinosis superficialis, calcinosis circumscripta, calcinosis universalis, and exoskeleton as illustrated in Table 1 [4, 9].

Since calcinosis is an uncommon condition, so far, there is no evidence-based standard treatment. Main strategies include the management of the underlying, predisposing chronic inflammation and, once the lesions have occurred, surgical removal [2, 5]. Few medical treatment options on calcinosis in mixed connective tissue disease have been successfully investigated so far [5, 7, 10].

Our report highlights a rare case of massive calcinosis of the elbow and describes the clinical symptoms, intraoperative findings, and the outcome six and 30 months postoperatively.

## 2. Case Report

### 2.1. History and Physical and Radiographic Examination

**Figure 1:**
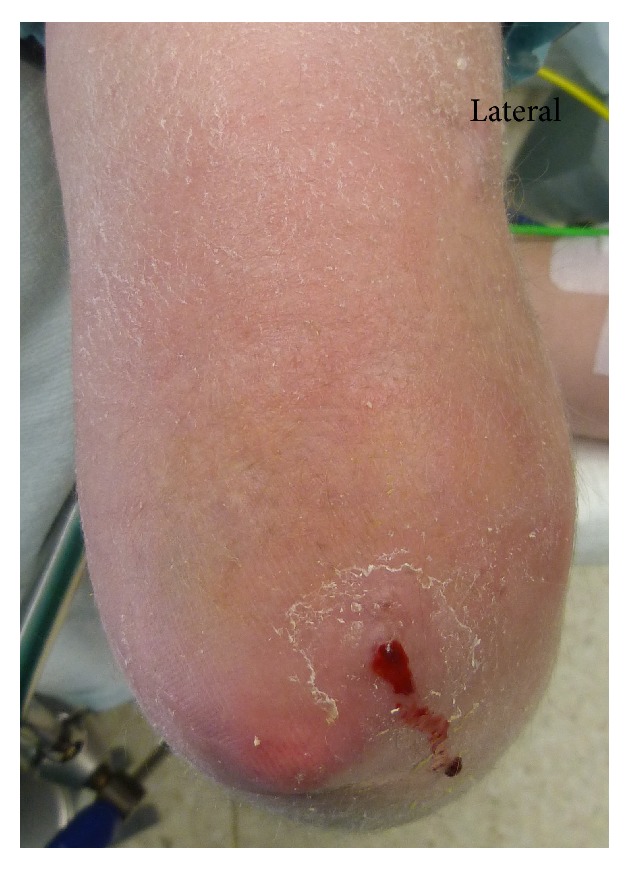
Right elbow of a 24-year-old woman with calcinosis universalis in juvenile Dermatomyositis; posterior view: fistula formation accompanied by erythema, swelling, and secretion of a blood tinged, serous secretion lateral to the olecranon.

**Figure 2:**
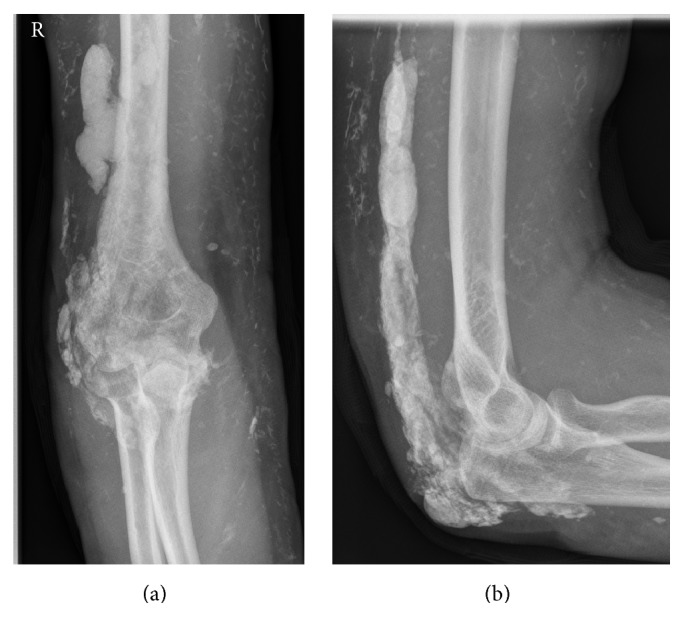
Standard elbow radiographs of a 24-year-old woman with calcinosis universalis in juvenile Dermatomyositis. (a) Anterior-posterior radiograph, (b) lateral radiograph.

**Figure 3:**
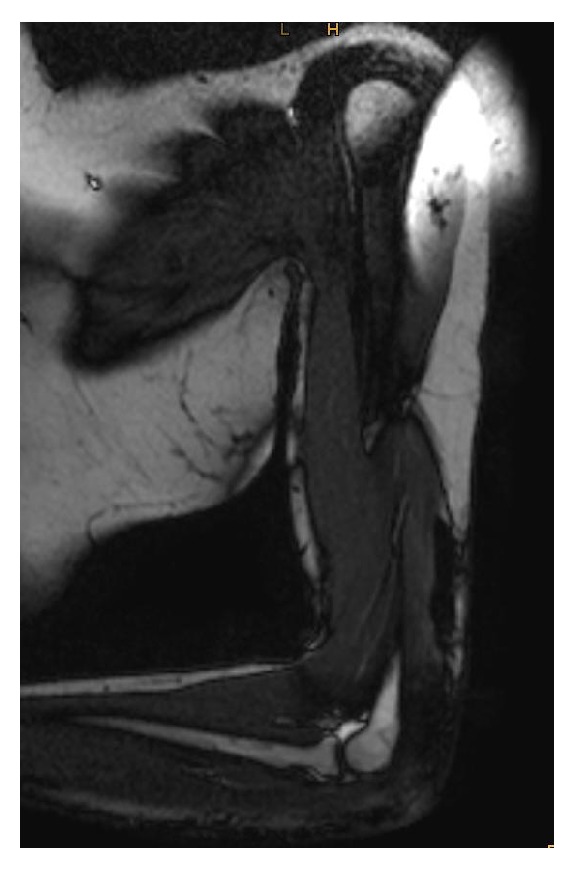
Sagittal T1 MRI scan of the right elbow of a 24-year-old woman with calcinosis universalis in juvenile Dermatomyositis with circumscribed areas of decreased signal intensity. No intra-articular effusion.

The patient, a 24-year-old woman, was first examined in January 2013. She reported that she had developed an inflammation and swelling of her right elbow over a period of two weeks without any history of trauma. Furthermore she complained about limited range of motion due to worsening arthralgia. Symptoms of systemic inflammation such as fever and chills were denied. Since the age of 15 the woman was suffering from an overlap syndrome with JDM and rheumatoid arthritis. At initial diagnosis ANA was positive and CK elevated. During the course of illness the woman had been treated with different combinations of high- and low-dose steroids, methotrexate, intravenous immunoglobulin, Azathioprine, and Hydroxychloroquinsulfate. At latest presentation to the rheumatologic clinic in June 2012 the laboratory analysis showed a C-reactive protein (CRP) of 0.06 mg/dL. Immune serology showed a rheumatoid factor of < 10 U/mL (reference value 0.0–14.0), a C3 of 40 mg/dL (reference value 75–140), a C4 of 17.2 mg/dL (10.0–34.0), and a CK of 106 U/L (0–170). ANA was below 80 (reference value 1: (0–160)), ENA screening was negative, and dsDNS antibodies were 0.3 U/mL (reference value 0.0–10.0). More recently the patient developed palpable subcutaneous indurations of the loins and the right elbow. Upon inspection there was no axis deviation of the right upper limb. But erythema, indurations, and swelling in vicinity of the posterior elbow were observed. Blood tinged, serous secretion lateral to the olecranon was noticed (Figure 1). The elbow joint was held in 90° flexed relieving posture. On clinical examination there was tenderness, an expressible white, crumbly matter, and a limited range of motion due to pain. Standard radiographs of the left elbow (Figures 2(a) and 2(b)) showed multiple diffuse larger opacities along the myofascial planer of the triceps tendon and smaller ones partly intramuscular. The initiated MRI ruled out an intra-articular affection (Figure 3). Laboratory analysis demonstrated an elevated CRP level of 8.9 mg/dL (reference value 0.0–0.8 mg/dL) and an accelerated erythrocyte sedimentation rate (ESR) of 67 mm/h (reference value < 25 mm/h), whereas parameters of renal retention were slightly low with a Creatinine level of 39 *μ*mol/L (reference value 45−84 *μ*mol/L). Further laboratory analysis showed a low vitamin D level of 6 ng/mL (reference value 20–40 ng/mL) and normal serum levels of phosphate with 1.09 mmol/L, of calcium with 2.53 mmol/L, of urea with 4.1 mmol/L, and of parathyroid hormone with 23 pg/mL as well as a normal activity of the alkaline phosphatase with 45 U/L.

The patient was admitted to our hospital for operative sampling, removal of the infected tissue, and broaching of the deposits.

### 2.2. Intraoperative Findings

**Figure 4:**
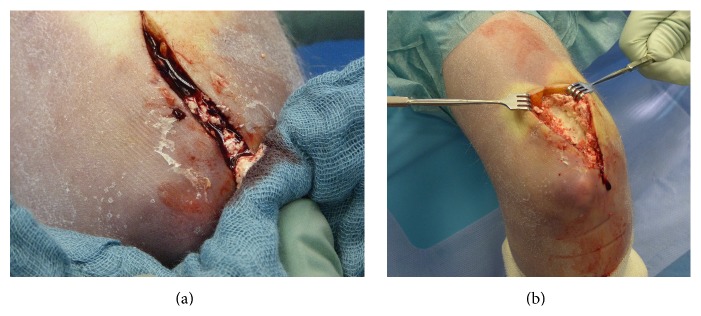
Intraoperative findings: (a) drainage of a white, crumbly paste via a sinus in calcinosis universalis associated with juvenile Dermatomyositis. (b) Subfascial preparation and broaching of the calcium deposits in the musculus triceps brachii and its tendon.

**Figure 5:**
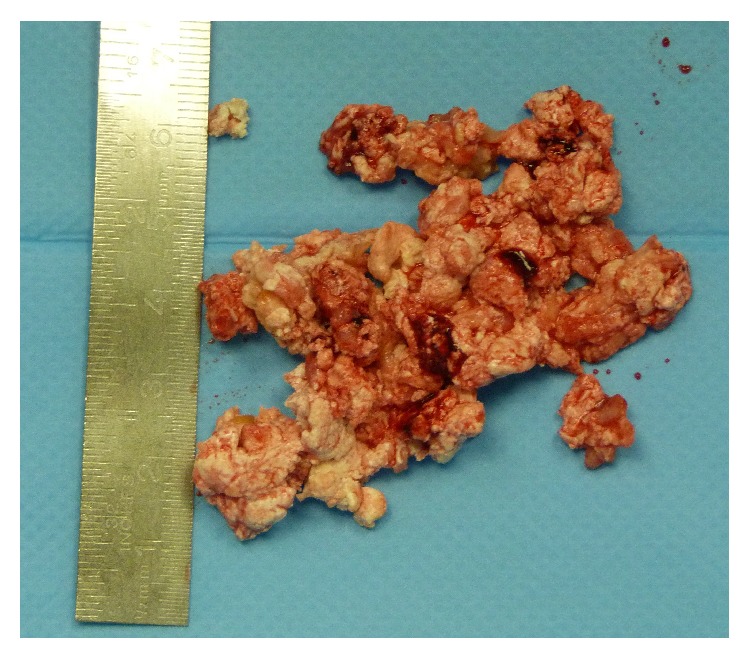
Broached material of the right elbow in a patient with calcinosis universalis associated with juvenile Dermatomyositis.

Approach was made by a seven-centimetre incision dorsal to the tendon of the musculus triceps brachii running from median to lateral of the olecranon. Subdermal preparation revealed a sinus tract formation from the lesion's site to epidermal, which was resected. On compression a white, crumbly deposit drained via the sinus (Figure 4(a)). A distinct bursitis olecrani was noticed. The operative exploration disclosed subfascial, calcific deposits of the musculus triceps brachii itself and its tendon (Figure 4(b)). Due to the risk of collateral tissue damage an incomplete broaching of the deposit was performed. Approximately ten millilitres of a white and yellowish crumbly paste with hard portions was removed (Figure 5). There was no macroscopical evidence for an infectious process. Microbiological and histological native samples were taken.

### 2.3. Postoperative Care and Follow-Up

After intraoperative specimens were taken, the patient was started on a cephalosporin during the stay in hospital.* Staphylococcus aureus* resistant to Penicillin G grew on the microbiological specimens. Postoperatively the patient was allowed physical therapy with limitation of flexion to 90°. There were no direct complications resulting from surgery such as impaired wound healing. On the eighth postoperative day CRP had returned to a normal value of 0.5 mg/dL and the patient was dismissed. Histological examination showed collagenous and fibrous tissue with calcific plaques, psammomas, and necrotic portions. Further there was an active granulomatous inflammation and formation of pseudocysts. The histopathological evaluation confirmed consistency with the clinical diagnosis of dystrophic calcinosis.

**Figure 6:**
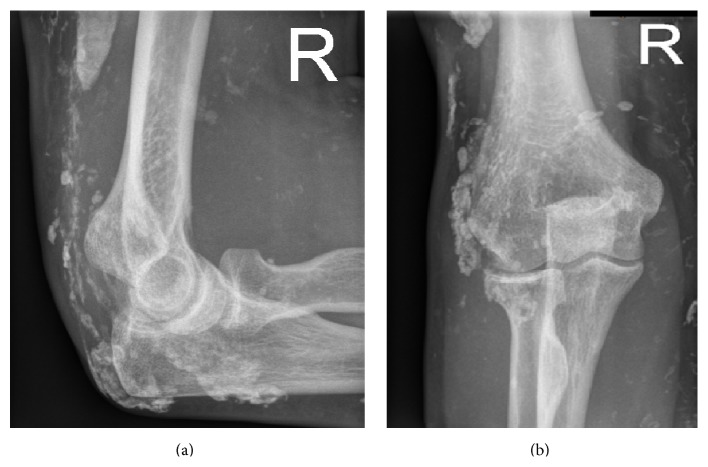
Standard elbow radiographs of a 24-year-old woman six weeks after cautious broaching of large calcific lesions in calcinosis universalis associated with juvenile Dermatomyositis of the musculus triceps brachii and its tendon plus resection of an ulcerating sinus on (a) anterior-posterior radiograph, (b) lateral radiograph.

**Figure 7:**
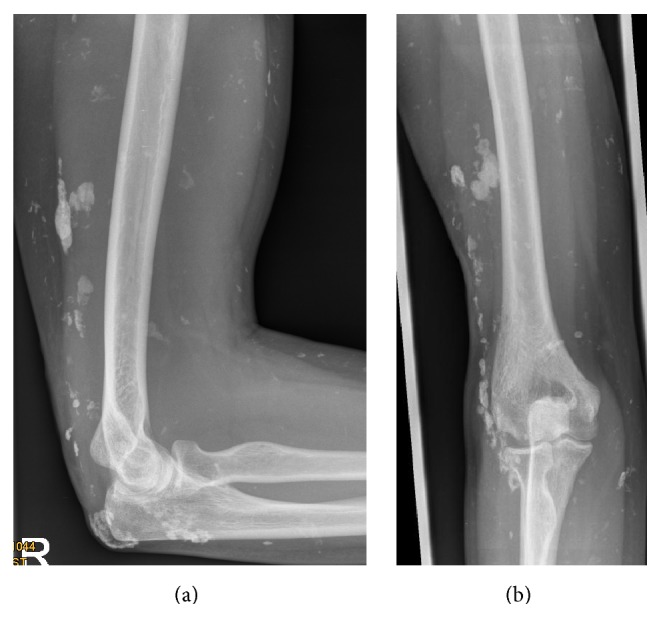
Standard elbow radiographs of a 24-year-old woman six months after cautious broaching of large calcific lesions in calcinosis universalis associated with juvenile Dermatomyositis of the musculus triceps brachii and its tendon plus resection of an ulcerating sinus on (a) anterior-posterior radiograph, (b) lateral radiograph.

**Figure 8:**
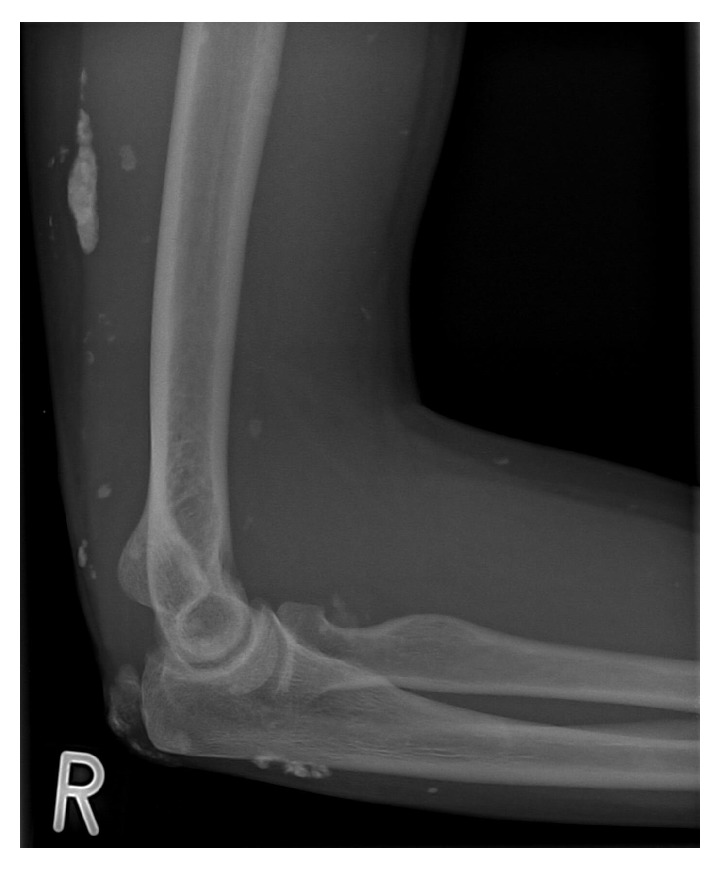
Standard lateral elbow radiograph of a 26-year-old woman 30 months after cautious broaching of large calcific lesions in calcinosis universalis associated with juvenile Dermatomyositis of the musculus triceps brachii and its tendon plus resection of an ulcerating sinus.

On six-week follow-up the patient presented free of pain. On clinical examination wound healing was unremarkable. Range of motion was completely restored. Standard radiographs of the left elbow (Figures 6(a) and 6(b)) showed a remarkably reduced calcium deposit without new formation in vicinity to the operation site. Laboratory analysis of CRP and ESR was unremarkable; six months postoperatively further spontaneous resorption had occurred (Figures 7(a) and 7(b)). After 30 months she was persistently free of pain; range of motion was free. The lateral X-ray showed further dissolving of the remaining calcific deposits (Figure 8).

## 3. Discussion

Calcinosis universalis is a rare but well known complication of JDM. However, little has been revealed about the pathogenesis. Chronic inflammatory state, in which affected tissue presents in JDM, is accepted as the aetiological background [5–7, 11]. This assumption is supported by elevated levels of macrophages and proinflammatory cytokines like IL-10 and IL-6 as well as TNF-alpha found in the calcific deposits. Serum levels of IL-1*β* are elevated in serum [7, 11]. Additionally, recent genotype investigations in JDM patients presenting with calcinosis have shown an association with a TNF-alpha-308A promoter polymorphism, which correlates with increased TNF-alpha production by peripheral blood mononuclear cells in the phenotype [4, 11]. The calcifications are characterised by calcium hydroxylapatite comparable to that in normal bone. However, the relative amount of mineral exceeds the amount in normal bone [4, 9]. So far, these investigations are especially of scientific value. None of these investigations have been performed in the reported case.

In general soft tissue calcifications are classified into 5 different subtypes depending on aetiology: metastatic, tumoral, idiopathic, calciphylactic, and, as in this case, dystrophic. JDM, scleroderma, and systemic lupus erythematosus are the most common entities leading to dystrophic calcifications. Laboratory testing of calcium, phosphorus, and parathyroid hormone serum levels in patients with dystrophic calcifications is usually unremarkable [6, 7]. So far, laboratory examinations are the most important diagnostic tool to rule out differential diagnoses, such as Hypervitaminosis D, nephropathy, hyperparathyroidism, and progressive osseous heteroplasia [6–8]. Although ossifying myositis and fibrodysplasia ossificans progressiva also present with unremarkable blood testing results, these differential diagnoses can be ruled out by the patient's history and physical examination: ossifying myositis is associated with posttraumatic haematoma, whereas fibrodysplasia ossificans progressiva develops in the first decade of life and typically presents with toe malformations and restrictive lung disease [8].

Although the clinical presentation is heterogenic, attempts to classify subtypes of dystrophic calcinosis were made. Earlier suggestions distinguished four subtypes of dystrophic calcinosis depending on affected tissue and the distribution pattern of the calcific deposits, which are calcinosis superficialis “with small, circumscribed nodules or plaques on the skin”; calcinosis circumscripta “with clumpy, discrete nodular subcutaneous masses, which usually occur near joints and interfere with movement”; calcinosis universalis “with sheet-like deposits in the intermuscular fascial planes”; and “exoskeleton” [4, 9]. But already in 1983 Bowyer et al. acknowledged that, in clinical practice, these subtypes fade continuously into each other. However, in the reported case the patient showed a classical distribution pattern of CU. She presented with a long history of inflammatory activity reflected by the refractory course of JDM [2, 5]. The period between diagnosis of JDM and manifestation of calcinosis was about 7 to 8 years, which is coherent with the observation by Bowyer et al. [9]. The periarticular location on the extensor aspect of the elbow, erythema, and painful limited range of motion are also considered common clinical features [5, 7]. Furthermore the patient developed infection and ulceration [2, 5]. The X-rays revealed the typical pattern of multiple subdermal, subfascial, and intramuscular calcifications [6]. Apart from the elevated CRP and ESR laboratory testing was unremarkable [7]. In synopsis a case of textbook-like presentation of CU is reported.

Although calcinosis in JDM is a common condition, there is no accepted standard treatment. Investigations on medical treatment in patients with calcinosis associated with JDM are rare. In 2008 Riley et al. treated five children aged six to nine years with a history of JDM between two and four years. These patients all suffered from symptomatic calcinosis and were treated over a period of 8 to 30 months with additional Infliximab, a monoclonal antibody against TNF-*α*. In four cases alleviation of clinical symptoms and reduction of radiological findings were observed. In the last case only clinical reduction is reported [10]. Marco Puche et al. reported a study of four children with calcinosis and JDM. The patients were treated with additional intravenous pamidronate, a nitrogen containing bisphosphonate. The drug was administered on three consecutive days every three months, resulting in significant decrease of calcinosis in three cases and one total reduction of calcinosis [12]. Further studies are mostly case reports testing Thalidomide [11], Diltiazem plus Prednisolone [13], and intravenous immunoglobulin [14]. To date the most crucial columns of treating calcinosis in JDM are hampering chronic inflammatory activity by antirheumatic drugs and, when calcinosis has developed, surgical treatment [2, 5]. Unfortunately, to our knowledge, there are no follow-up studies about patients with calcinosis and JDM. Once established, calcinosis remains difficult to treat. In our case, no complications were noted during the follow-up course. Complete restoration of range of motion and cure of infection were achieved. The patient was very satisfied with the postoperative result. After broaching with immediate clinical and radiographic improvement, further spontaneous resorption of the major deposit was observed and the result was enduring. This indicates that broaching of big symptomatic lesions in calcinosis especially in case of concomitant infection may be a viable way of treatment.

So far none of the treatment regimens, either operative or conservative, approved superior results. Further investigations on treatment of dystrophic calcinosis are therefore required.

## 4. Conclusions

This case report highlights a classical presentation of CU in JDM. Although refractory to medical therapy, surgical treatment showed a satisfactory outcome. To date there are promising developments for drug regimes, but in this severe case surgery has proved to be a reliable treatment option in symptomatic calcinosis universalis. Further investigations on conservative and operative treatment options are required.

## Figures and Tables

**Table 1:** Proposed classification of dystrophic calcinosis by Bowyeret al. [9].

Type	Distribution	Typical impairment
Calcinosis superficialis	Small, circumscribed nodules and plaques on the skin	Rarely interferes with function and is usually painless

Calcinosis circumscripta	Clumpy, discrete nodular subcutaneous masses, which usually occur near joints	Generally in the proximal muscle groups with severe pain and functional limitations

Calcinosis universalis	With sheet-like deposits in the intermuscular fascial planes	Almost always associated with significant discomfort and limitation of movement

Exoskeleton	A lacy, reticular pattern of calcification extending throughout the body at the level of subcutaneous tissue	Generalised erythroderma, with friable skin and ulcerations
